# Letter to the editor on exercise and immune competence: using pre‐clinical findings to inform the design of clinical studies

**DOI:** 10.1113/EP092745

**Published:** 2025-04-02

**Authors:** Amit Hagar

**Affiliations:** ^1^ Indiana University Bloomington Indiana USA

**Keywords:** CD4+, endurance exercise, hypoxia, immune avoidance, immunosupressors, solid tumor, TME, VEGF

1

I would like to comment on a recent paper on exercise and intratumour infiltration of immune cells (Thomsen et al., 2026) that found no difference in infiltration of CD8^+^ and CD3 cells into in vivo solid tumours' Tumor Microenvironment's (TMEs) after 8 weeks of exercise between exercised and control subjects.

While I commend the attempt to use pre‐clinical findings to inform the design of clinical studies in human subjects, I would like to highlight the challenges that await such attempts.

The authors of the above paper cite my own pre‐clinical study as evidence for the general effects of exercise on immune competence. However, in my study we, too, found no difference in CD8^+^ infiltration into the TME after exercise training between exercised and sedentary (mice) groups; the difference we found was in the infiltration of the FoxP3^+^ Treg cells – or rather, in the ratio between CD8^+^ and FoxP3^+^ Treg cells. Indeed, the mechanism we hypothesized in that study that underlies the slowing down of solid tumour growth with (endurance) exercise was that the latter increases the host adaptation to hypoxia thus decreases the tumour's ability to recruit these immunosuppressor cells via vascular endothelial growth factor signalling by postponing the metabolic shift to glycolysis and by degrading hypoxia‐inducible factor 1‐α (see e.g. Westendorf et al., 2017). As a result, I was not surprised to see no difference in CD8 or CD3 cells in their (human) study after training. I wish that the authors would have tested the exercised TMEs for immunosuppressor FoxP3^+^ Treg cells as well, and not only the CD8^+^ cells for which no difference was expected anyway.

This brings me to the second point about exercise and immune response. Exercise is a very general term, and the type and dosage of exercise matter a lot in exercise oncology, especially when conducting pre‐clinical trials, the findings of which are expected to be translatable to humans. One reason people are looking for immune cells in the TME after exercise may be the 2016 report on the infiltration of NK cells into the TME from Pedersen's team (Pedersen et al., 2016). However, that report has two major differences from the pre‐clinical study I conducted. First, rather than on a metabolic shift, it relies on a different mechanism (interleukin‐6 receptor trans‐signalling, or more generally, a fever‐like effect on the trafficking of immune cells into a tissue). Second, such a fever‐like effect, unfortunately, cannot be immediately translated into humans, as it involves an exercise regimen that may be above the human threshold (but which mice can nevertheless naturally follow). This was the reason we developed an endurance training protocol that would train mice to run like humans (and avoided using the voluntary wheel altogether), so that any results we would get would be translatable to humans, who do not run like mice. See also Melo & Hagar (2019).

I do hope more human studies on the effect of endurance exercise on solid tumour patients will be done in the future, which could validate the mechanism of post‐training adaptation to hypoxia and the suppression of immunosuppressor recruitment to the TME. Prostate cancer seems to be a preferred target as Standard of Care in that space often includes ‘active surveillance’ (also called ‘watchful waiting’), which permits the necessary exercise intervention period.

## AUTHOR CONTRIBUTIONS

Sole author.

## CONFLICT OF INTEREST

The author declares he has no conflicts of interest.
